# The Impact of Psoriasis and Sexual Orientation on Mental and Physical Health Among Adults in the United States

**DOI:** 10.1016/j.jaad.2021.07.066

**Published:** 2021-08-08

**Authors:** Matthew D. Mansh, Amy Mulick, Sinéad M Langan

**Affiliations:** 1 Department of Dermatology, University of Minnesota, Minneapolis, Minnesota; 2 London School of Hygiene and Tropical Medicine, London, United Kingdom; 3 St. John’s Institute of Dermatology, London, United Kingdom

## To the Editor

Psoriasis is a chronic inflammatory disorder that causes physical disfigurement and impacts mental health and quality of life.^1^ Sexual minorities (SMs) report higher rates of mental health and chronic medical issues,^2,3^ and other chronic skin diseases, such as acne vulgaris, have been found to disproportionately affect mental health among SMs.^4^ This study assesses the impact of sexual orientation on the relationship between psoriasis, mental health and physical health among U.S. adults.

We conducted a secondary analysis of population-based, cross sectional data from the 2003-2006 and 2009-2014 National Health and Nutrition Examination Surveys, including heterosexual and SM (lesbian, gay, bisexual or “something else”) participants aged 18-59 years. Among all participants (by psoriasis status) and in analyses stratified by psoriasis status (by sexual orientation), we calculated prevalence rates and prevalence odds ratios using unadjusted and multivariable-adjusted logistic regression analyses for primary outcomes including clinical depression (based on a Patient Health Questionnairre-9), mental healthcare utilization, overall health, frequent physical distress, and frequent mental distress. Interaction analyses were conducted between SM identity and psoriasis status. All statistical analyses were weighted and performed using STATA version 16.1. This study using publicly-available, de-identified data was exempt from institutional review board review.

The study included 14,932 heterosexual (371 with psoriasis, 14,561 without psoriasis) and 1,004 SM (30 with psoriasis, 974 without psoriasis) participants (Supplemental Figure 1; Table I). In multivariable analyses, participants with psoriasis compared to those without had higher odds of clinical depression, mental healthcare utilization, frequent physical distress, and frequent mental distress. Among those without psoriasis, sexual orientation was associated with all primary outcomes. Among those with psoriasis, SMs compared to heterosexuals had higher odds of clinical depression, reporting fair or poor health and frequent physical distress. Interaction analyses may have been underpowered, but we found an interaction between SM identity and psoriasis for mental healthcare utilization (p=0.04) (Table II). Data on psoriasis severity and medical comorbidities were not included in multivariable analyses, but are available by SM identity among participants with psoriasis in Supplemental Table I.

This study suggests that SMs with psoriasis report poorer mental and physical health than heterosexuals with psoriasis. In particular, we found SMs with psoriasis had nearly 4-fold higher odds of reporting symptoms of clinical depression, frequent physical distress and poor overall health. Despite these differences, SMs with psoriasis were no more likely than heterosexuals with psoriasis to report receiving mental healthcare, indicating that physician interventions might be lacking. High baseline rates of mental^2^ and physical health^3^ issues among SMs likely contribute to these differences. Study strengths include the use of a nationally-representative sample. Limitations include self-reported data and the small number of SMs with psoriasis.

Futures studies are needed to better understand factors contributing to the mental and physical health impact of psoriasis among sexual minorities and the potential for intersectionality with other minority identities (e.g. race/ethnicity). Increasing routine collection of sexual orientation,^5^ mental health and the quality-of-life impact of psoriasis will empower dermatologists to provide the high-quality, patient-oriented care these populations need and deserve.

## Supplementary Material

Supplemental Figure 1

Supplemental Table 1

## Figures and Tables

**Table I:** Demographic and clinical characteristics by psoriasis status and by sexual orientation stratified by psoriasis status among adults aged 18-59 years, NHANES 2003-2006 & 2009-2014

	All Participants (N=15,936)^b^		No Psoriasis ^a^ (N=15,535) ^b^		Psoriasis ^a^ (N=401) ^b^	
Characteristic^c^	No Psoriasis^a^ (N=15,535)^b^ % (SE)	Psoriasis^a^ (N=401)^b^ % (SE)	Heterosexual (N=14,561)^b^ %(SE)	Sexual Minority (N=974)^b^ %(SE)	Heterosexual (N=371)^b^ %(SE)	Sexual Minority (N=30)^b^ %(SE)
**Age, mean**	38.6 (0.2)	42.1 (0.7)	38.7 (0.2)	37.0 (0.6)	42.1 (0.7)	42.3 (2.1)
**Age, categorical**						
18-29 years	27.9 (0.7)	15.4 (3.0)	27.4 (0.7)	35.0 (2.3)	16.1 (2.2)	5.2 (3.0)
30-39 years	23.2 (0.5)	25.5 (3.0)	23.2 (0.5)	22.6 (1.5)	24.5 (3.1)	39.5 (11.0)
40-49 years	25.6 (0.6)	26.8 (3.4)	25.9 (0.6)	21.1 (1.6)	27.1 (3.5)	22.9 (8.6)
50-59 years	23.3 (0.6)	32.4 (2.9)	23.4 (0.6)	21.3 (2.0)	32.3 (3.1)	32.5 (10.1)
**Sex**						
Male	50.2 (0.4)	48.3 (2.9)	50.8 (0.4)	40.9 (2.2)	48.6 (3.0)	45.1 (11.1)
Female	49.8 (0.4)	51.7 (2.9)	49.2 (0.4)	59.1 (2.2)	51.4 (3.0)	54.9 (11.1)
**Race/Ethnicity**						
Non-Hispanic White	66.4 (1.6)	80.6 (2.1)	66.6 (1.6)	63.1 (2.3)	81.0 (2.1)	75.3 (7.5)
Non-Hispanic African	12.0 (0.8)	5.9 (1.0)	11.8 (0.8)	14.4 (1.5)	5.8 (1.1)	7.7 (4.1)
Hispanic	14.9 (1.1)	8.4 (1.5)	15.0 (1.2)	13.4 (1.5)	8.0 (1.5)	14.7 (6.0)
Other	6.7 (0.4)	5.1 (1.1)	6.6 (0.4)	9.1 (1.1)	5.3 (1.1)	2.4 (1.7)
**Education Level**						
Less than High School	15.6 (0.7)	12.6 (1.6)	15.5 (0.7)	17.1 (1.5)	12.4 (0.17)	15.4 (6.6)
High School or GED	22.4 (0.7)	20.8 (2.4)	22.6 (0.7)	19.2 (1.6)	21.1 (2.5)	16.0 (8.4)
Some college or associates degree	32.6 (0.7)	30.9 (2.9)	32.5 (0.7)	34.0 (1.8)	30.1 (2.8)	43.2 (10.8)
College degree	29.4 (1.1)	35.7 (3.1)	29.4 (1.1)	29.7 (2.4)	36.4 (3.2)	25.5 (9.6)
**Usual source for care^d^**						
Yes	81.5 (0.4)	86.6 (2.1)	81.5 (0.4)	81.4 (1.4)	87.2 (2.1)	77.3 (8.2)
**Insured^e^**						
Yes	77.0 (0.7)	80.4 (2.6)	77.4 (0.8)	72.5 (1.9)	80.0 (2.7)	85.1 (5.3)
**Body Mass Index**						
Underweight (<20.0)	5.4 (0.3)	2.8 (1.0)	5.3 (0.3)	6.7 (0.9)	2.9 (1.0)	1.3 (1.3)
Normal Weight (20.0 -24.99)	28.1 (0.7)	22.2 (2.2)	28.0 (0.7)	29.8 (1.7)	21.2 (2.1)	35.9 (10.4)
Overweight (25.0-29.9)	31.9 (0.6)	33.7 (2.9)	32.3 (0.6)	25.7 (1.6)	34.6 (2.9)	21.5 (8.9)
Obese (>=30.0)	34.6 (0.7)	41.3 (3.3)	34.5 (0.7)	37.7 (1.8)	41.3 (3.4)	41.3 (9.6)
**Smoking History**						
Never	56.1 (0.7)	45.9 (3.0)	56.8 (0.8)	46.8 (2.1)	44.9 (2.9)	59.2 (10.1)
Former	19.1 (0.6)	30.4 (2.6)	19.2 (0.6)	16.7 (1.7)	31.4 (2.8)	15.0 (7.3)
Current	24.8 (0.7)	23.8 (2.7)	24.0 (0.7)	36.5 (1.8)	23.6 (2.8)	25.9 (9.0)

a Psoriasis status based on response to “Have you ever been told by a healthcare provider that you had psoriasis?”

b Unweighted sample sizes for reference

c Reporting percentages or means (+/- standard error) based on the weighted sample.

d Healthcare access status based on response to “Is there a place that you usually go when sick or need advice about your health?”

e Insurance coverage status based on response to “Are you covered by insurance or some other kind of health care plan?”

**Table II:** Mental and physical health outcomes by psoriasis status and by sexual orientation in analyses stratified by psoriasis status among adults aged 18-59 years, NHANES 2003-2006 & 2009-2014

			Stratified Analysis				
	All Participants		No Psoriasis		Psoriasis		
	No Psoriasis	Psoriasis	Heterosexual	Sexual Minority	Heterosexual	Sexual Minority	p-interaction^g^
**Clinical Depression^a^**							
PHQ-9 Score, Mean (SE)	3.0 (0.1)	4.0 (0.3)	2.9 (0.1)	4.8 (0.3)	3.9 (0.3)	6.0 (1.5)	
Clinical Depression, % (SE)	7.6 (0.3)	11.2 (2.2)	7.0 (0.3)	18.0 (1.9)	9.9 (2.1)	30.5 (9.1)	
Unadjusted OR (95% CI)	1.0 (Ref)	1.53 (1.01-2.39)	1.0 (Ref)	2.94 (2.26-3.82)	1.0 (Ref)	3.98 (1.31-12.08)	0.59
Adjusted OR, (95% CI)^f^	1.0 (Ref)	1.51 (1.01-2.48)	1.0 (Ref)	2.45 (1.87-3.21)	1.0 (Ref)	3.75 (1.26-11.13)	0.58
**Mental Health Care Utilization in Last 12 Months^b^**							
Seen Mental Health Care Provider, % (SE)	8.9 (0.4)	13.2 (2.2)	8.3 (0.4)	19.1 (1.9)	13.2 (2.4)	11.7 (4.2)	
Unadjusted OR (95% CI)	1.0 (Ref)	1.55 (1.07-2.25)	1.0 (Ref)	2.63 (2.02-3.42)	1.0 (Ref)	0.87 (0.25-3.06)	0.09
Adjusted OR (95% CI) ^f^	1.0 (Ref)	1.45 (1.01-2.14)	1.0 (Ref)	2.40 (1.84-3.13)	1.0 (Ref)	0.78 (0.23-2.63)	0.04
**Overall Health^c^**							
Poor or Fair Health, % (SE)*	14.8 (0.5)	16.6 (2.3)	14.5 (0.5)	19.3 (1.5)	15.2 (2.1)	36.9 (9.5)	
Unadjusted OR (95% CI)	1.0 (Ref)	1.15 (0.84-1.57)	1.0 (Ref)	1.41 (1.16-1.72)	1.0 (Ref)	3.25 (1.29-8.18)	0.08
Adjusted OR (95% CI)^f^	1.0 (Ref)	1.17 (0.84-1.65)	1.0 (Ref)	1.36 (1.09-1.69)	1.0 (Ref)	4.05 (1.27-12.88)	0.08
**Frequent Physical Distress^d^|**							
Physical Unhealthy Days in Last Month, Mean (SE)	3.1 (0.1)	4.1 (0.5)	3.1 (0.1)	4.1 (0.4)	3.8 (0.5)	9.4 (2.3)	
Frequent Physical Distress, % (SE)	8.9 (0.4)	13.2 (2.2)	8.6 (0.4)	13.2 (1.5)	11.8 (2.2)	33.7 (9.9)	
Unadjusted OR (95% CI)	1.0 (Ref)	1.56 (1.04-2.33)	1.0 (Ref)	1.61 (1.23-2.10)	1.0 (Ref)	3.80 (1.42-10.16)	0.09
Adjusted OR (95% CI)^f^	1.0 (Ref)	1.54 (1.01-2.17)	1.0 (Ref)	1.47 (1.11-1.93)	1.0 (Ref)	3.76 (1.19-11.91)	0.10
**Frequent Mental Distress^e^**							
Mental Unhealthy Days in Last Month, Mean (SE)	4.2 (0.1)	5.5 (0.5)	4.1 (0.1)	6.8 (0.5)	5.4 (0.5)	6.7 (2.0)	
Frequent Mental Distress, % (SE)	12.5 (0.5)	17.9 (1.9)	11.9 (0.4)	22.2 (1.9)	17.6 (2.0)	22.3 (7.1)	
Unadjusted OR (95% CI)	1.0 (Ref)	1.53 (1.19-1.96)	1.0 (Ref)	2.11 (1.69-2.63)	1.0 (Ref)	1.34 (0.50-4.31)	0.43
Adjusted OR (95% CI)^f^	1.0 (Ref)	1.44 (1.10-1.88)	1.0 (Ref)	1.92 (1.54-2.38)	1.0 (Ref)	1.46 (0.42-5.15)	0.61

a Clinical depression score based on responses to a 9-item Patient Health Questionnaire (PHQ-9) with possible scores ranging between 0 to 27. Clinical depression was defined as a PHQ-9 score of 10 or greater.

b Mental health care utilization based on a positive response to “During the past 12 months, have you seen or talked to a mental health professional such as a psychologist, psychiatrist, psychiatric nurse or clinical social worker about your health?”

c Overall poor or fair health status defined a response of either “poor” or “fair” to “Would you say your health in general is (1) excellent (2) very good (3) good (4) fair, or (5) poor?”

d Physically unhealthy days based on response to “Thinking about your physical health, which includes physical illness and injury, for how many days during the past 30 days was your physical health not good?” Frequent physical distress was defined as reporting ≥ 14 physically unhealthy days in the last 30 days.

e Mentally unhealthy days based on response to “Thinking about mental health, which includes stress, depression, and problems with emotions, for how many days during the past 30 days was your mental health not good?” Frequent mental distress was defined as reporting ≥ 14 mentally unhealthy days in the last 30 days.

f Adjusted odds ratios based on multivariable logistic regression analyses controlling for sexual orientation, age, sex, race, education level, healthcare access (usual source of care), health insurance status, body mass index, and smoking history.

g P-value for the interaction term between sexual orientation and psoriasis in a multivariable logistic regression model controlling for sexual orientation, psoriasis status, age, sex, race, education level, healthcare access (usual source of care), health insurance status, body mass index, and smoking history.
