# Supplementary figures and images for: The Impact of Psoriasis and Sexual Orientation on Mental and Physical Health Among Adults in the United States
Source: J Am Acad Dermatol. Author manuscript; Available in PMC 2022 Jul 1. (PMC7612892; doi:10.1016/j.jaad.2021.07.066)

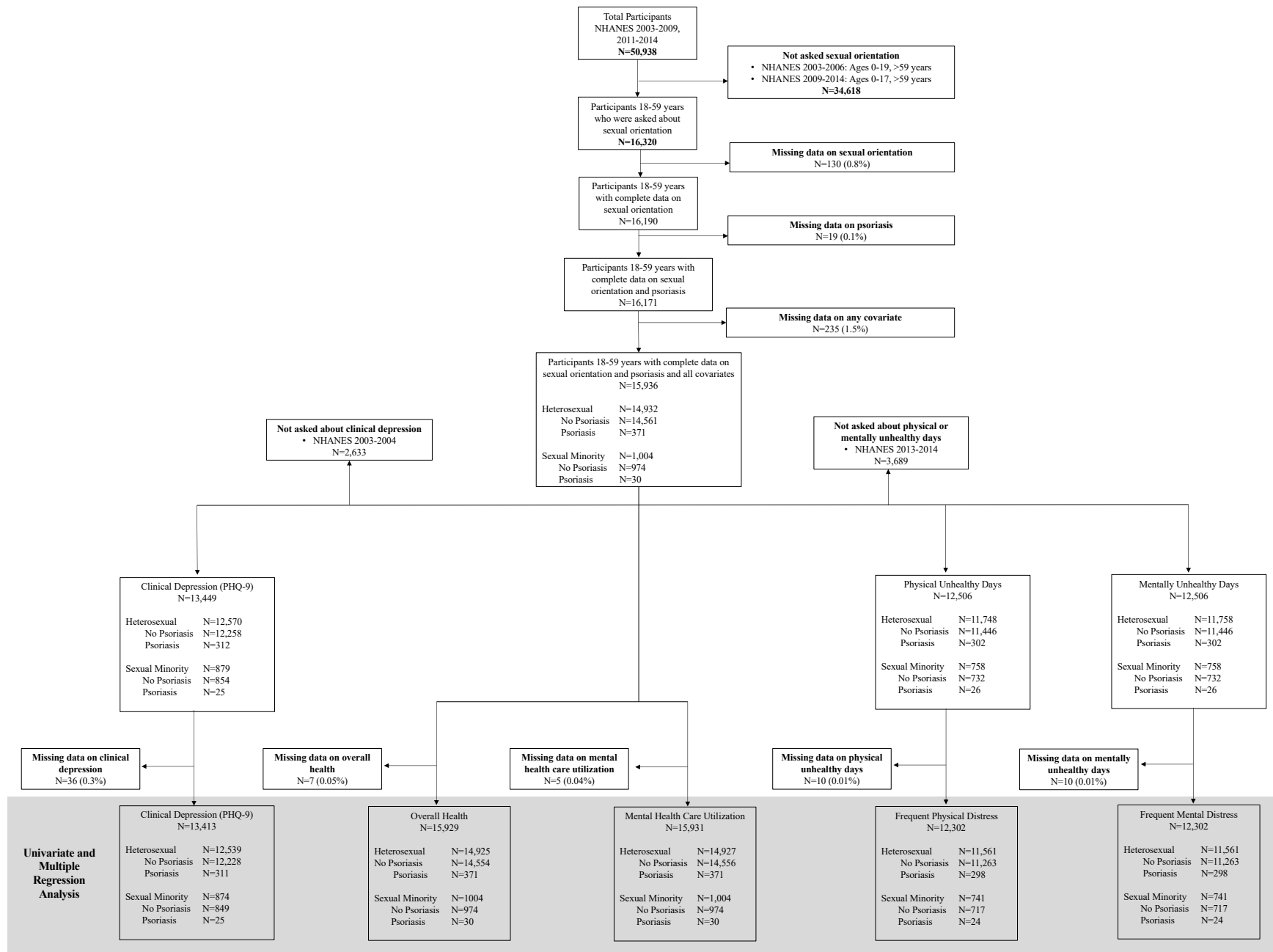

Supplement: Supplemental Figure 1 [file EMS135991-supplement-Supplemental_Figure_1.pdf]
