# Supplementary material for: The Impact of Psoriasis and Sexual Orientation on Mental and Physical Health Among Adults in the United States
Source: J Am Acad Dermatol. Author manuscript; Available in PMC 2022 Jul 1. (PMC7612892; doi:10.1016/j.jaad.2021.07.066)
Supplement: Supplemental Table 1 [file EMS135991-supplement-Supplemental_Table_1.docx]

**Supplemental Table I**. Psoriasis severity and medical co-morbidities by sexual orientation among adults aged 18–59 years with psoriasis, NHANES 2003-2006 & 2009-2014

| **Psoriasis Severity**^a^ | Heterosexual  (N=287),  % (SE) | Sexual Minority  (N=23),  % (SE) |
| --- | --- | --- |
| Little or No Psoriasis | 53.5 (4.3) | 60.8 (13.0) |
| 1-2% Body Surface Area Involvement | 24.2 (3.4) | 27.4 (11.2) |
| ≥ 3% Body Surface Area Involvement | 22.4 (3.6) | 11.8 (7.9) |
| **Medical Co-morbidities** | Heterosexual  (N=370),  % (SE) | Heterosexual  (N=30),  % (SE) |
| **History of cardiovascular disease**^b^ | 3.9 (1.1) | 1.7 (1.7) |
| **History of diabetes**^c^ | 4.1 (1.0) | 12.6 (6.9) |
| **History of arthritis**^d^ | 32.9 (3.1) | 40.5 (11.4) |

^a^ Psoriasis severity based on response to “Do you currently have (1) Little or no psoriasis, (2) Only a few patches (that could be covered by one or two palms of your hand), (3) Scattered patches (that could be covered between three and ten palms of your hand), or (4) Extensive psoriasis (covering large areas of the body, that would be more than ten palms of your hand)?”. Analyses limited to respondents from NHANES 2003-2006 & 2011-2014 including 287 heterosexual and 23 sexual minorities with psoriasis. Data not collected in 2009/2010 survey years.

^b^ Cardiovascular disease defined as a positive response to questions asking if the individual was ever told by a doctor or other health professional that they had (1) congestive heart failure, (2) coronary heart disease, (3) angina, (4) heart attack, or (5) stroke.

^c^ Diabetes defined as positive response to “Other than during pregnancy, have you ever been told by a doctor or health professional that you have diabetes or sugar diabetes?”

^d^ Arthritis defined as positive response to “Have you ever been told by a doctor or health professional that you had arthritis?”.
